# Diffuse Supravalvular Aortic Stenosis: Surgical Repair in Adulthood

**DOI:** 10.4061/2009/976190

**Published:** 2009-11-04

**Authors:** Giovanni Ferlan, Claudio De Pasquale, Concetta Losito, Annalisa Fiorella, Nicola Marraudino, Francesco Tunzi

**Affiliations:** Department of Cardiac Surgery, University of Bari Medical School, Piazza Giulio Cesare 11, Bari 70122, Italy

## Abstract

We present the case of a 54-year-old woman in which a diffuse congenital supravalvular aortic stenosis (SVAS) was associated with a severe aortic valve incompetence and heavy calcification of the aortic annulus. Repair consisted in resection of the ascending aorta, patch augmentation of the hypoplastic aortic root and annulus, placement of a 20 mm Dacron tubular graft (Vascutek, Renfrewshire, UK) and aortic valve replacement with a mechanical prosthesis (Sorin, Turin, Italy). Follow-up echocardiography demonstrated normal prosthetic valve function and a postoperative three-dimensional computed tomographic scan showed a normal shape of the reconstructed ascending aorta.

## 1. Introduction

Congenital supravalvular aortic stenosis (SVAS), either in form of a discrete or a diffuse narrowing of the ascending aorta, is the least common type of left ventricular outflow obstruction.Peripheral pulmonary artery stenosis, coronary lesions, abnormalities of the aortic valve leaflets may also be present in patients with SVAS. Calcification of the aortic annulus is uncommon. Moreover, survival beyond the 4th decade is rare because of the secondary effects of the disease on the left ventricle, the coronary circulation and the aortic valve.

## 2. Case History

**Figure 1:**
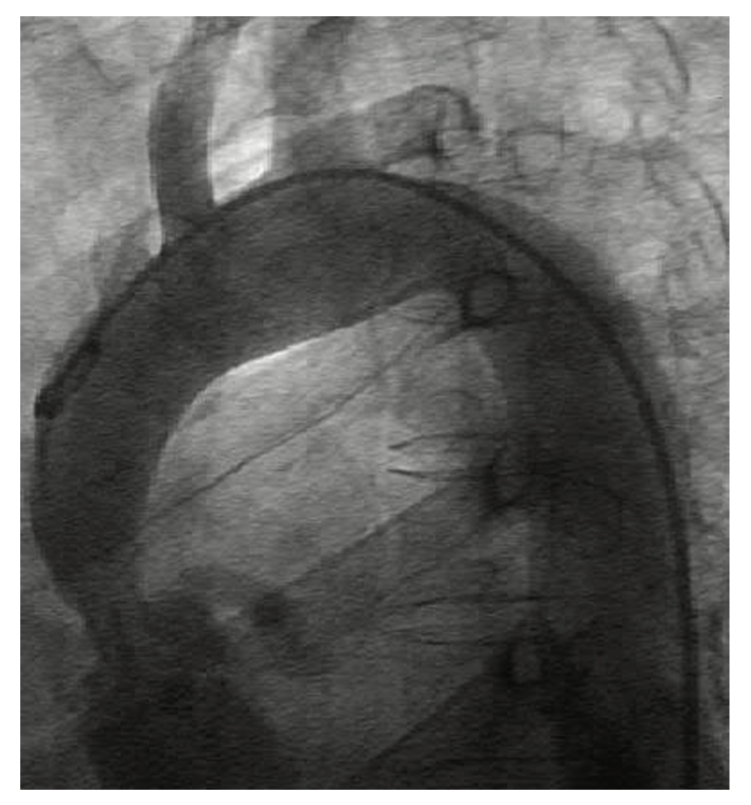
Preoperative angiography.

A 54-year-old woman presented with a 1-year-history of deteriorating exertional dyspnea. Auscultation revealed a grade IV-V/VI systolic ejection murmur. Transthoracic echocardiography demonstrated moderate concentric left ventricular hypertrophy, hypoplasia of the ascending aorta, and aortic valve annulus (diameter: 16 mm) with peak gradients of 175 mmHg across the ascending aorta. The aortic valve was largely incompetent, due to the presence of a very small and displastic left coronary cusp; the aortic annulus was heavily calcified. Angiography (Figure 1) showed a severe hypoplasia of the proximal segment of the ascending aorta (minimum inner diameter: 11 mm) and a 2/4 aortic incompetence; a mild stenosis of the isthmic region was also present, with a pressure gradient of about 35 mmHg The coronary ostia were enlarged and the right coronary and the left main coronary were dilated and tortuous, but the coronary vessels were free of any obstructive disease. Pulmonary vasculature was normal.

**Figure 2:**
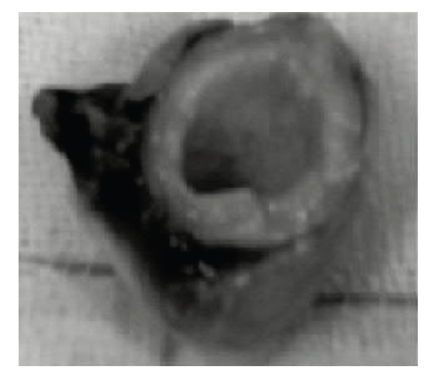
Intraoperative ascending aorta specimen.

After benzodiazepine premedication, induction and maintenance of general anesthesia were accomplished with midazolam hydrochloride (0,1 mg/Kg) and fentanyl citrate (5*γ*/Kg) infusions. Care was taken to maintain always the mean arterial pressure over 80 mm Hg; if necessary, norephrine was eventually administered e.v. The operation was performed with moderate hypothermic cardiopulmonary bypass. After cardioplegic cardiac arrest, the aorta was incised transversely 1 cm above the sinotubular junction. The native ascending aorta showed a very thick wall (4 mm), see Figure 2. The aortic valve had three leaflets, which were free of adherence to the sino-tubular area and did not cause any impairement to the filling of the coronary arteries; however, the left coronary leaflet was hypoplastic, and this could explain the severe aortic incompetence found. Abundant calcifications were present in the annulus and in the commissural areas. The aortic sinuses and the coronary ostia were free of calcification, as well as the anterior mitral leaflet. After excision of the aortic valve, the annulus was meticulously debrided of calcific deposits. According to the height (1.47 m), weight (45 kg) and body surface area of the patient (1.34 m^2^), a 17S Sorin mechanical valve (estimated orifice area:cmq. 1.70 m^2^; EOA/BSA: 1.26 m^2^) was selected. To allow an enlargement of both the aortic annulus and the supravalvular ascending aorta as well, the aortotomy incision was prolonged toward the tip of the commissure between the left and the noncoronary leaflets. The incision was stopped immediately above the hinge of the anterior mitral leaflet and a tear-drop shaped patch was then inserted with a continuous 4-0 Prolene suture, as described by Manouguian [1]. The enlarged aortic root could now accomodate the selected mechanical valve and a 20 mm Dacron tubular graft to replace the ascending aorta.

**Figure 3:**
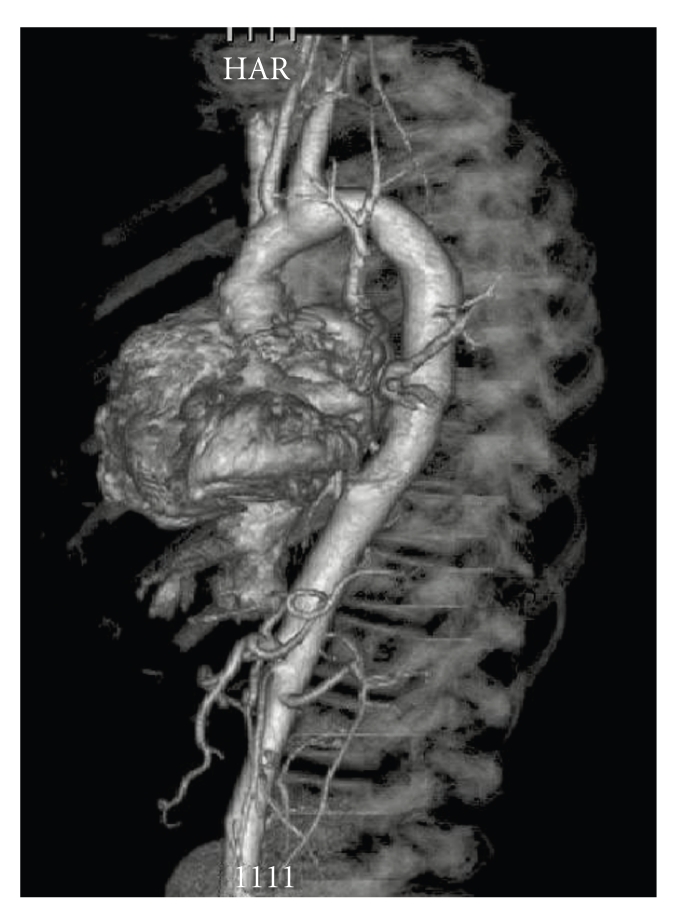
Postoperative CT angiography.

The microscopic examination of the excised aortic wall showed medial thickening with an increased number of hypertrophied smooth muscle cells, increased collagen content, and reduced elastic tissue in form of broken and disorganized elastin fibers, as reported by Stamm et al. [2]. Postoperative transesophageal echocardiography showed normal function of the mechanical valve and a peak gradient of 25 mmHg The patient was discharged on the seventh post-operative day. Three months later, a CT-scan demonstrated a normal aspect of the ascending aorta (Figure 3).

## 3. Discussion

The prognosis of patients with SVAS is related to the severity of aortic stenosis which, in turn, influences the age of presentation [3]: accordingly, a 30-year survival is predictable in only 12% of pediatric patients with severe SVAS. Structural changes of the coronary vessels, either related to a well-known elastin gene defect [4], or to the effects of chronic elevated prestenotic systolic pressure, which promotes dilatation and tortuosity of coronary vessels or premature arteriosclerosis [5], have been often reported in patients with SVAS and may cause impairement of coronary circulation. Furthermore, severe ventricular hypertrophy can lead to a critical perfusion mismatch, chronical subendocardial ischemia, and chronic left ventricular disfunction. Both of these factors account for the elevated risk of sudden death in patients with SVAS and a severe aortic stenosis.

Our case, probably a sporadic form of SVAS, is interesting because of the advanced age of presentation of symptoms and the long-term survival, despite the severity of the left ventricular outflow obstruction. Calcification of the aortic annulus is rarely reported in these patients and may represent a surgical challenge, particularly when an aortic annulus enlargement is needed. To enlarge the aortic root, two techniques have been described, which both require an extension of the conventional aortotomy incision through the commissure between the left and noncoronary sinuses [1], or through the midpoint of the noncoronary sinus across the mitral valve [6], and the insertion of a teardrop shaped Dacron patch cut to the required dimensions. In our patient, we chose the first technique, which moreover seemed to us safer than a composite root replacement because it avoided any manipulation of the enlarged and fragile coronary ostia.

In our experience, too, an ECG-gated computed tomography has proved to be an excellent diagnostic tool in evaluating such patients [6].
